# Pharmacovigilance Study of Anticancer Drugs in a Tertiary Care Teaching Hospital in North India: A Retrospective Study

**DOI:** 10.7759/cureus.44984

**Published:** 2023-09-10

**Authors:** Raj Kumar, Amita Jindal, Pardeep Garg, Amandeep Kaur, Sumir Kumar, Rakesh Tilak Raj, Simrandeep Singh

**Affiliations:** 1 Pharmacology and Therapeutics, Guru Gobind Singh Medical College and Hospital, Baba Farid University of Health Sciences, Faridkot, IND; 2 Radiation Oncology, Guru Gobind Singh Medical College and Hospital, Baba Farid University of Health Sciences, Faridkot, IND; 3 Dermatology, Guru Gobind Singh Medical College and Hospital, Baba Farid University of Health Sciences, Faridkot, IND; 4 Dermatology, Government Medical College, Guru Nanak Dev Hospital, Amritsar, IND; 5 Oncology, Guru Gobind Singh Medical College and Hospital, Baba Farid University of Health Sciences, Faridkot, IND

**Keywords:** modified hartwig and siegel scale, modified schumock and thornton scale, molecular targeted drugs, taxanes, chemotherapy adverse effects, dermatological side effects, drug-related adverse reactions, anticancer agents, who-umc causality assessment scale

## Abstract

Introduction: Anticancer agents are responsible for a majority of adverse drug reactions (ADRs) in cancer patients. ADR reporting with anticancer drugs is very rare in India due to the lack of awareness and knowledge about the Pharmacovigilance Programme of India. Hence, this study was done to assess the pattern of ADRs with anticancer agents in cancer patients and to increase awareness about ADR monitoring among healthcare professionals.

Materials and methods: This is an observational, retrospective and non-interventional study conducted in an ADR monitoring centre (AMC) in Govt. Guru Gobind Singh Medical College and Hospital, Faridkot, Punjab, North India. Voluntarily reported ADR forms with anticancer drugs as suspected drugs over a period of seven years from January 2016 to December 2022 were analyzed. Various parameters were analyzed, which include demographic details of the patients, type of ADR, department reporting ADR and suspected drug. Causality assessment, severity assessment and preventability assessment were done according to the World Health Organization Uppsala Monitoring Centre (WHO-UMC) scale, modified Hartwig and Siegel scale and modified Schumock and Thornton scale, respectively.

Results: The maximum numbers of ADRs were reported in the age group of 41-60 years (68.29%) and in females (59.75%). The maximum number of ADRs was reported with the use of taxanes (docetaxel and paclitaxel) (24.39%), targeted drugs (geftinib, imatinib, bortezomib, bevacizumab, rituximab and pazopanib) (24.39%) and platinum co-ordination complexes (cisplatin, oxaliplatin and carboplatin) (17.07%). Majority of the ADRs reported were shivering and ADRs on the skin. Majority of the ADRs were probable (64.70%), mild in nature (85.29%), definitely preventable (45.58%) and probably preventable (45.58%).

Conclusion: ADR monitoring is needed to increase the outcome of anticancer drug treatment in cancer patients. The quality of treatment in cancer patients can be improved through the timely management of these ADRs. It is a need of the present era to inform healthcare professionals about the Pharmacovigilance Programme to increase the reporting of ADRs due to anticancer drugs.

## Introduction

Cancer is a leading cause of deaths worldwide, accounting for nearly 10 million deaths in 2020 [1]. The crude rate of incidence of cancer in India is 100.4 per one lac. In India, one in nine people are likely to develop cancer in his/her lifetime [2].

Many different anticancer drugs are available in the market, which can be used alone or in combination to treat different types of cancers. Although anticancer drugs treat different cancer effectively, they are also responsible for many adverse drug reactions (ADRs). Some of these ADRs are mild and manageable, while some are serious and require hospitalization [3].

The World Health Organization (WHO) defined ADR as “a response which is noxious and unintended and which occurs at doses normally used in humans for the prophylaxis, diagnosis, or therapy of disease or for the modification of physiological function” [4]. In India, the prevalence of ADRs with anticancer drugs is 10-12% [5]. However, ADR reporting is very rare in India due to the lack of knowledge on the Pharmacovigilance Programme of India among healthcare professionals [6].

Hence, this study was done to assess the pattern of ADRs with anticancer agent(s)/medicine(s) and to create awareness in healthcare professionals about the Pharmacovigilance Programme.

## Materials and methods

This is an observational, retrospective, non-interventional study conducted in the ADR monitoring centre (AMC) of Govt. Guru Gobind Singh Medical College and Hospital, a tertiary care teaching institute in Faridkot, Punjab, North India. In this study, voluntarily reported ADR forms received at the AMC over a period of seven years from January 2016 to December 2022 were analyzed. Only the ADR forms in which the suspected drug was an anticancer medicine were included in the study.

Various parameters were evaluated, such as demographic details of the patients, type of ADR, department of reporting ADRs and suspected medicine. The WHO Uppsala Monitoring Centre (WHO-UMC) scale was used to assess the causality [7]. The modified Hartwig and Siegel scale was used to assess the severity [8], while the modified Schumock and Thornton Scale was used to assess the preventability [9].

Numbers and percentages were used to express the obtained data.

## Results

A total of 82 ADR reporting forms were analyzed in which the suspected drug was an anticancer drug. The maximum number of ADRs were reported in the middle-age group and in females (Table 1).

**Table 1:** Demographic details of the patients

		Number	Percentage
Gender	Male	33	40.2%
	Female	49	59.75%
Age (years)	≤40	14	17.07%
	41-60	56	68.29%
	≥ 61	12	14.63%

**Figure 1:**
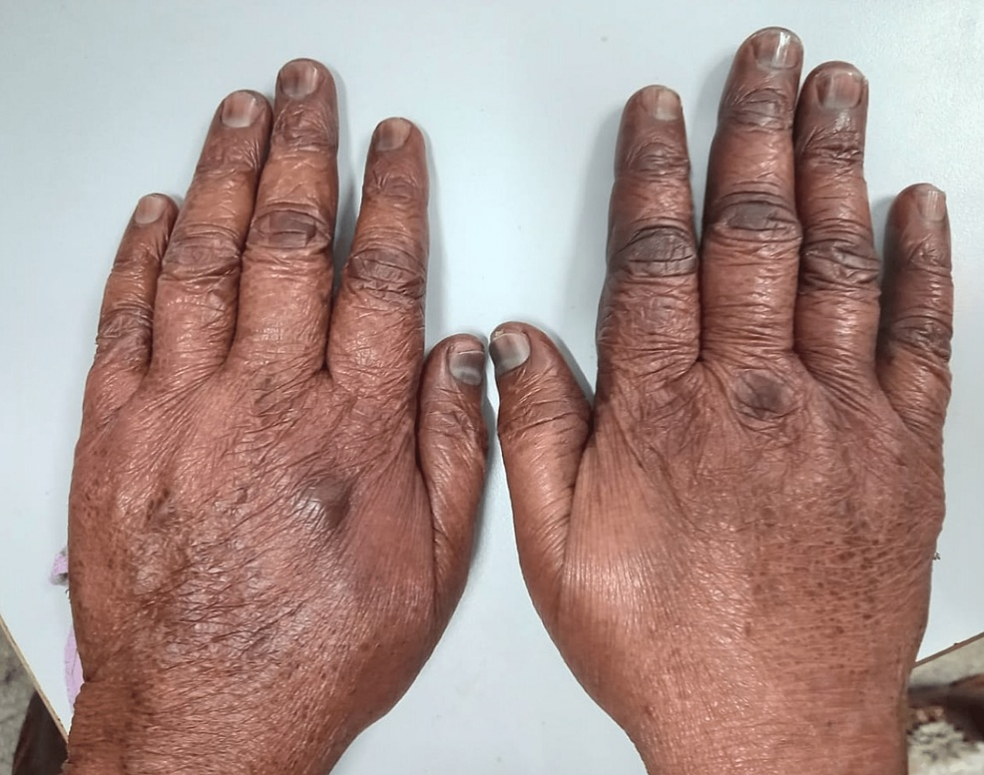
Paclitaxel-induced ADR. Image showing nail plate pigmentation and melanonychia along with hyperpigmentation of the knuckles and xerosis in both hands.

In this study, 81.70% ADRs were reported with a single anticancer agent (Table 2). The maximum number of ADRs were reported with the use of taxanes (docetaxel and paclitaxel) (24.39%) and targeted drugs (gefitinib, imatinib, bortezomib, bevacizumab, rituximab and pazopanib) (24.39%) and platinum co-ordination complexes (cisplatin, oxaliplatin and carboplatin) (17.07%). Some of the ADRs were also reported with the use of antimetabolites, alkylating agents, topoisomerase-2 inhibitor, vinca alkaloids, doxorubicin and immunosuppressive drugs, such as cyclosporine. Majority of the ADRs that were reported with the use of taxanes were seven cases of shivering, six cases of breathlessness and five cases with ADRs on the skin (bluish pigmentation, alopecia, itching, photodermatitis, mucosal erosions and nail deformities) (Figure 1). Targeted drugs were suspected in seven cases of shivering and six cases with ADRs on the skin (rashes, erythematous lesions and itching). Four cases of dizziness, four cases of shivering and three ADRs related to the skin were reported with the use of platinum co-ordination complexes (Table 2).

**Table 2:** ADRs reported with a single anticancer agent 5FU: fluorouracil, BP: blood pressure

S. no.	Anticancer drug	Number (*n*)	Percentage	Type of ADRs
1.	Taxanes	20	24.39%	
a.	Docetaxel	11	13.41%	Breathlessness (5), bluish pigmentation and alopecia (1), body ache (1), body ache and shivering (1), itching (1), photodermatitis (1), shivering (1)
b.	Paclitaxel	9	10.97%	Chills and shivering (5), mucosal erosions (1), nail discoloration and deformity of nails (1), fever and weakness (1), nausea and breathlessness (1)
2.	Targeted drugs	20	24.39%	
a.	Rituximab	11	13.41%	Chills and shivering (7), chest pain (1), nausea and headache (1), severe headache (1), pain in the throat and hoarseness of voice (1)
b.	Geftinib	3	3.65%	Rashes (2), handfoot syndrome (1), itching (1), erythematous lesions (1)
c.	Imatinib	3	3.65%	Rashes (2), erythematous lesions (1)
d.	Bortezomib	1	1.21%	Rashes
e.	Bevacizumab	1	1.21%	Rise in BP
f.	Pazopanib	1	1.21%	Erythematous lesions
3.	Platinum co-ordination complexes	14	17.07%	
a.	Cisplatin	7	8.53%	Diziness (3), maculopapular rashes (1), chills and shivering (1), diarrhea (1), breathlessness (1)
b.	Oxaliplatin	4	4.87%	Maculopapular rashes (1), chills and shivering (1), redness of the face and nausea (1), redness of the face and shivering(1)
c.	Carboplatin	3	3.65%	Chills and shivering (1), dizziness (1), itching (1)
4.	Antimetabolites	6	7.31%	
a.	5FU	2	2.43%	Rise in BP (1), nausea, shivering and redness of face (1)
b.	Capecitabine	3	3.65%	Purpuric skin rashes (1), alopecia (1), itching and eryythematous lesions(1)
c.	Methotrexate	1	1.21%	Vomiting
5.	Alkylating agents	3	3.65%	
a.	Chlorambucil	1	1.21%	Nail pigmentation
b.	Dacrabazine	1	1.21%	Rashes
c.	Temozolomide	1	1.21%	Erythematous lesions, swelling on the hands and feet
6.	Topoisomerase-2 inhibitor (etoposide)	1	1.21%	Severe pain in the body
7.	Vinca alkaloids (vincristine)	1	1.21%	Vomiting
8.	Antibiotics (doxorubicin)	1	1.21%	Vomiting
9.	Immunosupressive drugs (cyclosporine)	1	1.21%	Shivering
	Total	67	81.7%	

Some of the ADRs were also reported with the use of anticancer drug combinations (18.29%), as shown in Table 3.

**Table 3:** ADRs reported with the combinations of anticancer agents

S. no.	Drug combination	Number	Percentage	Type of ADRs
1	Paclitaxel + carboplatin	3	3.65%	Abdominal pain and stools (1), numbness of the hands and feet (1), nausea, dizziness and dry mouth (1)
2	5-fluorouracil + oxaliplatin	2	2.43%	Redness of face and shivering (1), phlebitis on both arms (1)
3	Paclitaxel+ cisplatin	2	2.43%	Dizziness, vertigo and constipation
4	Cyclophosphamide + adriamycin + docetaxel	1	1.21%	Backpain
5	Cyclophosphamide + doxorubicin + rituximab + vincristine	1	1.21%	Shivering
6	Paclitaxel + docetaxel	1	1.21%	Dizziness
7	5-fluorouracil + doxorubicin + cyclophosphamide	1	1.21%	Vomiting
8	Docetaxel + carboplatin	1	1.21%	Erythematous lesions
9	Gemcitanib + cisplatin	1	1.21%	Swelling on the hands and foot
10	Doxorubicin+ bleomycin+ vinblastine+ dacarbazine	1	1.21%	Ulcers in the mouth
11	Gemcitabine+ capecitabine	1	1.21%	Extreme weakness
	Total	15	18.29%	

Most of the ADRs were reported from the oncology department of the hospital (n=62, 75.60%), followed by the skin and venereal diseases (VD) (n=16, 19.51%) and medicine department (n=4, 4.87%).

**Figure 2:**
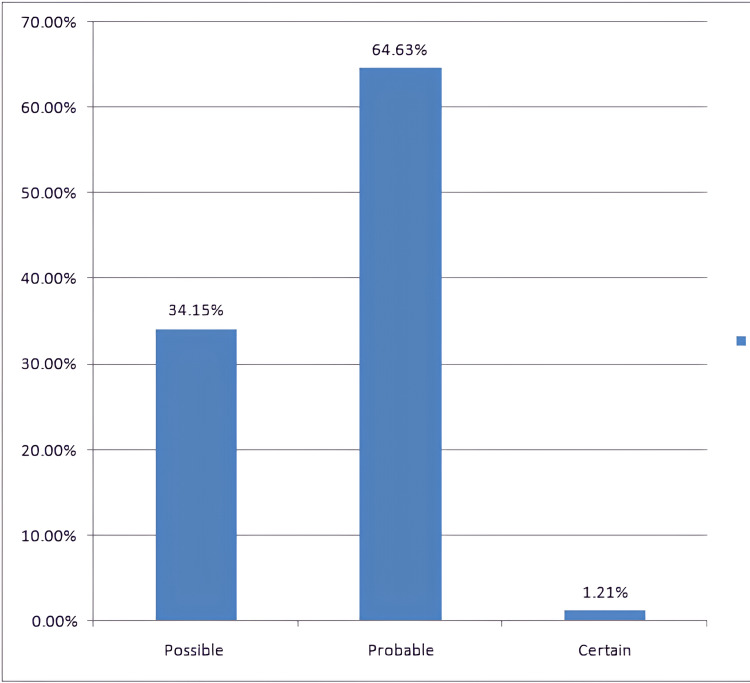
Causality assessment of ADRs World Health Organization Uppsala Monitoring Centre (WHO-UMC) causality assessment scale [7]

Causality assessment using the WHO-UMC scale showed that 64.63% (n=53) of the ADRs were probable, 34.15% (n=28) possible and 1.21% certain (Figure 2).

**Figure 3:**
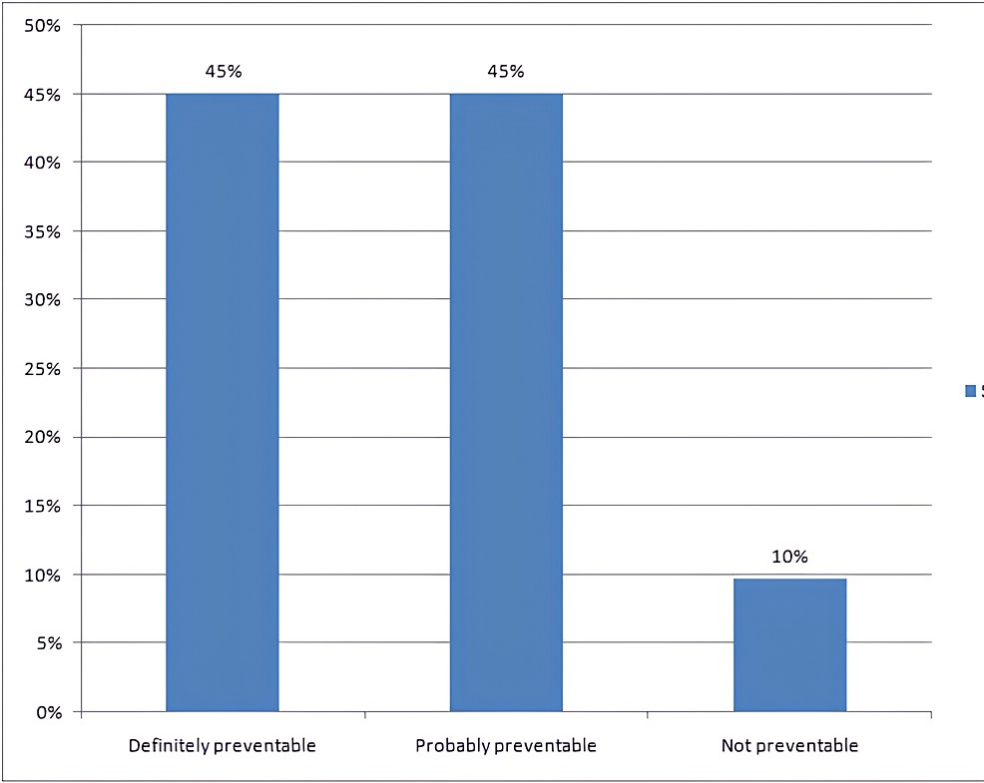
Preventability assessment Modified Schumock and Thornton preventability assessment scale [9]

The modified Schumock and Thornton scale for the assessment of preventability showed that majority of the ADRs were definitely preventable and probably preventable (n=37, 45.12% each) (Figure 3).

**Figure 4:**
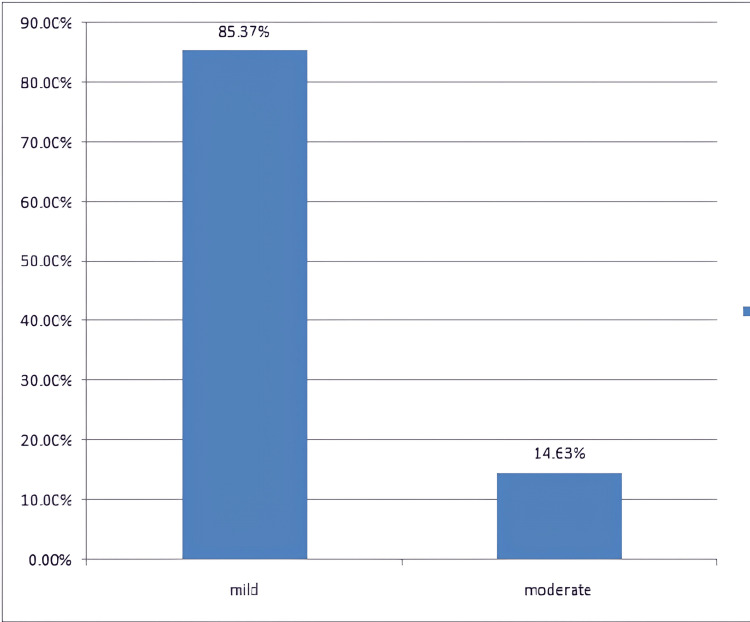
Severity assessment scale Modified Hartwig and Siegel severity assessment scale [8]

The modified Hartwig and Siegel scale for the assessment of severity of the ADRs showed that majority of the ADRs were mild in nature (85.29%), as shown in Figure 4.

## Discussion

ADR forms in which the suspected drug was any anticancer agent were collected and analysed in the present study. The maximum number of ADRs was reported in females. In similar studies [10,11], females also reported the maximum number of ADRs in comparison to males.

Majority of the ADRs were seen in the middle-age group in our study, which is similar with another study [11].

In this study, the major ADRs reported were shivering and ADRs on the skin. By contrast, in a study conducted by Singh et al. [12], alopecia was the major ADR reported with anticancer drugs, followed by nausea, vomiting and numbness.

In our study, majority of the ADRs were reported with the use of anticancer drugs, such as taxanes, targeted drugs and platinum co-ordination complexes. Some of the ADRs were also reported with the use of anti-cancer drug combinations. In a study conducted by George et al. [13], rituximab (targeted drug) was reported in one of the common suspected anticancer agents. Other studies [14,15] had shown that platinum co-ordination complexes reported the maximum number of ADRs similar to our study.

While assessing the causality in the present study, majority of the ADRs were probable in nature, which is similar to other studies [13,15]. While assessing the preventability, we noticed that majority of the ADRs were definitely preventable and probably preventable. This finding is concordant with the studies conducted by Sunny et al. [16] and Swathi et al. [17] that also noticed that majority of the ADRs were definitely preventable in nature. 

In this study, majority of the ADRs were mild in nature. This finding is also in concordant with other studies [14,15].

We also collected ADR forms from over a seven-year period. However, there were significantly less number of ADRs reported with anticancer drugs as the suspected drug. There can be multiple reasons for this. This may be due to the under-reporting of ADRs with anticancer drugs due to heavy work load or lack of awareness of the Pharmacovigilance Programme or sometimes healthcare professionals prefer to report only those ADRs that are not expected from a particular drug. There is a need to inform healthcare professionals about the Pharmacovigilance Programme to increase the reporting of ADRs with anticancer agents. With the help of ADR reporting, we can improve the outcome of anticancer drug treatment in cancer patients.

## Conclusions

Most of the ADRs were observed in the middle-age group and in females. The major ADRs reported were shivering and ADRs on the skin. Majority of the ADRs were reported with the use of taxanes, targeted drugs and platinum co-ordination complexes. The maximum number of ADRs were probable, mild, definitely and probably preventable. We conclude that by analyzing the pattern and assessment of ADRs with anticancer medicines, the quality of treatment in cancer patients can be improved by the timely management of these ADRs.
